# Socio-economic accounting of inequalities in excess weight: a population-based analysis

**DOI:** 10.1186/s12889-023-15592-0

**Published:** 2023-04-20

**Authors:** Paolo Candio, Fiorella Parra Mujica, Emma Frew

**Affiliations:** 1 Department of Economics and Management, University of Trento, Trento, Italy; 2 Centre for Economics of Obesity, Institute of Applied Health Research, University of Birmingham, Birmingham, UK; 3 Erasmus School of Health Policy and Management (ESHPM), Erasmus University Rotterdam, Rotterdam, Netherlands; 4 Nuffield Department of Population Health, Health Economics Research Centre (HERC), University of Oxford, Oxford, UK

**Keywords:** Excess weight, Inequality, Socio-economic status, Accounting

## Abstract

**Background:**

The prevalence of excess weight has been increasing globally in the last decades, affecting disproportionally adults from low socio-economic backgrounds and putting undue pressure on health systems and societal resources. In England, tackling unfair and unjust health inequalities is at the heart of national public health policy, and a prerequisite for enabling these decision makers to set policy priorities is an understanding of the prevalence and determinants of excess weight inequalities in their local population.

**Methods:**

We conducted both pooled (England) and regional-level (nine regions: North-East, North-West, Yorkshire and Humber, East Midlands, West Midlands, East of England, London, South East and South West) analyses of individual level data from a nationally representative sample of adults (*N* = 6,387). We used the Corrected Concentration Index (CCI) to measure absolute inequalities in excess weight across three dimensions of socio-economic deprivation: neighbourhood-level deprivation, occupational status and educational qualification. We used a Shapley decomposition method to evaluate their relative contribution to inequality.

**Results:**

At a national level, all three dimensions of socio-economic deprivation were found to be positively associated with excess weight across the adult population, as measured by the CCI, with educational qualification ranking first [CCI: -0.090, *p* < 0.01], closely followed by neighbourhood-level deprivation [CCI: -0.050, *p* < 0.01]. Large variation was found between regions and genders, with inequality being either considerably higher or exclusively patterned among women. The strongest independent factor contributing to excess weight inequalities was having a long-lasting limiting illness, especially among women and towards the right tail of the excess weight spectrum. Heterogeneous patterns of contribution across the excess weight spectrum were found, however age played a dominant role toward the left tail of the distribution.

**Conclusions:**

While socio-economic inequalities in excess weight exist in the English adult population, our findings underscore the importance of considering multiple dimensions of deprivation and the unique needs of different populations when developing policies to address overweight and obesity. Targeted interventions for adults with overweight and obesity with long-lasting illnesses and women can generate both short-term and long-term economic benefits, by reducing healthcare costs and increasing workforce productivity.

**Supplementary Information:**

The online version contains supplementary material available at 10.1186/s12889-023-15592-0.

## Background

Excess weight is a major challenge for many countries around the world. By 2021, around two billion adults (39%) had excess weight – that is, a body mass index (BMI) of at least 25, a third of which (13%) being affected by obesity (BMI of at least 30) [1]. In Western countries, these figures are even starker [2]. In England, currently around two-thirds of adults are at least overweight and 31% live with obesity [3], causing inevitably profound human and economic consequences.

Overweight and obesity generate societal costs, that is costs that affect individuals while having a wider impact on society. Societal costs of overweight and obesity can be classified broadly into three categories: direct, indirect and intangible costs [4].Direct costs include the costs of medical treatment, such as hospitalization, medication, and doctor visits. These are relevant as excess weight, and more so obesity, are associated with chronic health conditions including type II diabetes [5], cardiovascular [6] and respiratory disease [7] and cancer [8]. Indirect costs include decreased productivity, increased absenteeism from work, and higher healthcare costs related to the strain that overweight and obesity causes on the healthcare system [9]. In the United Kingdom, the annual direct cost from treating health complications related to population obesity has been estimated to be over £6 billion in 2014, with wider societal costs of £27 billion [10]. Finally, intangible costs include the non-monetary impacts of obesity, such as decreased quality of life, reduced self-esteem and stigma [11].

The prevalence and rates of increase of excess weight are not spread equally across societies [12, 13], and evidence has accumulated for a positive relationship between excess weight and socio-economic deprivation in developed economies [14]. In this context, deprivation refers to a lack of material (financial and non-financial) resources and opportunities which impairs living conditions. At an individual level, living in deprivation—that is being at a low socio-economic status (e.g., low educational attainment or income level)—involves facing multiple barriers to achieving a good quality of life, including limited access to healthcare and healthy food options, inadequate housing, fewer opportunities for physical activity, poor education, and higher levels of stress and anxiety. At the local level, areas of high deprivation are characterized by having fewer resources and facilities that support healthy lifestyles, such as health care facilities, green spaces, and healthy and affordable food outlets.

In England, a cross-sectional analysis of 2010–2012 data from the Understanding Society database [15] explored income-related inequalities in adiposity and found that ‘pro-rich’ inequalities were evident, especially among women [16]. Aligning with these findings, a 2012 report indicated that this relationship could hold for a range of socio-economic measures, including occupational status, where the prevalence of obesity among unskilled workers (35.2%) was estimated to be twice as large as that among professionals (18.2%) [17]. Another cross-sectional study from the same time period focused on regional variation in adult excess adiposity in England and found that these differences were more evident towards the right tail of the distribution and that a neighbourhood-obesogenic environment contributed to excess adiposity independently from individual lifestyle and occupational status [18].The existing unfair and unjust health inequalities have motivated the recent ‘levelling up’ policy agendas, which are at the core of the public health discourse, often filtering down to local-level jurisdictions [19]. A prerequisite for enabling public health policy makers to address this issue is understanding the scale and nature of the problem. This requires gauging the current prevalence and understanding the determinants of excess weight in local populations and among equity-relevant subgroups.

### Motivation for this study

Previous research on this topic has mostly focused at the country level and this evidence might not reflect specific regional characteristics, as the role of socio-economic factors and demographic compositions, in particular gender, may vary markedly between regions. This is important to consider for informing regional policies and avoid a widening of the existing inequalities [20]. Furthermore, if we consider excess weight as a spectrum ranging from overweight to morbid obesity, then a person’s risk for developing obesity can depend on an array of individual, social and economic factors [21]. Understanding how these factors independently contribute across this spectrum is important to inform the design of tailored interventions, that is focused on overweight or on obesity-related outcomes.

In providing up-to-date, country and regional-level estimates of excess weight inequalities in England, this study aimed to answer the following two research questions:

1. How do different measures of socio-economic deprivation pattern excess weight across the English and regional-level adult population?
2. What are the key contributing factors to these socio-economic inequalities?

## Methods

### Study design and settings

We conducted both pooled (England) and regional-level (nine regions: North-East, North-West, Yorkshire and Humber, East Midlands, West Midlands, East of England, London, South East and South West) analyses of individual level data from a nationally representative sample of adults. We explored gender-related heterogeneity and tested for statistical associations in terms of excess weight, as well as focusing only on obesity.

### Data sources

We analysed data from the 2019 wave of the Health Survey for England [22] (HSE), an annual repeated cross-sectional survey of adults from a nationally representative sample of private households. This survey provides information on respondents’ socio-demographic characteristics (e.g., age, gender, and socio-economic status) and their health and lifestyle status. The 2019 edition comprised 8,205 adults with a valid (interviewer-assisted) BMI measurement, who were recruited from 9,612 addresses selected at random from 534 postcode sectors.

To adjust for survey non-response, we applied a set of weights available within HSE for the different elements of the survey. For 18.6% of the targeted survey respondents BMI measurement values were missing. To correct for this potential source of selection bias, we applied an inverse probability weighting method, in line with the approach used for HSE [23]. To further adjust for imbalances between the sampling quotas and the final survey samples, post-stratification weights were constructed using the inverse probability weighting-derived adjustments and a ranking procedure [24].

### Outcome variable

Excess weight was the dependent variable. This was derived from the respondents’ BMI score across five categories according to the current classification for adults [25]: healthy weight, BMI >  = 18.5 and < 25 (no excess weight); overweight, BMI >  = 25 and < 30; obesity I, BMI >  = 30 and < 35; obesity II BMI >  = 35 and < 40 and obesity III, BMI >  = 40.

### Indicators of socioeconomic deprivation

Three measures of socio-economic deprivation were considered: the Index of Multiple Deprivation (IMD) quintile, occupational status, and educational qualification. The English IMD measures relative levels of deprivation in 32,844 small areas (neighbourhoods with an average of approximately 1,500 residents or 650 households) and is organised across seven domains of deprivation which are combined and weighted (income, 22.5%; employment 22.5%; health deprivation and disability, 13.5%; education, skills training 13.5%; crime 9.3%; barriers to housing and services 9.3%; living environment 9.3%) [26]. All neighbourhoods in England are then ranked in deprivation order. We included the IMD as a relevant proxy for measuring socio-economic status-related inequalities in overweight and obesity, because individuals living in deprived areas (as indicated by the IMD) may have comparatively limited access to healthy food options and safe places to exercise, which can contribute to higher rates of overweight and obesity [16–18].

Occupation and education are also commonly used as proxies for socioeconomic status because they provide a comprehensive picture of an individual’s relative economic and social position. Individuals in low-status occupations or with lower levels of education may have less access to health information and resources, leading to unhealthy lifestyle choices that can contribute to overweight and obesity. The National Statistics Socio-economic classification was used as the measure of occupational status. This composite measure is constructed based on aspects of work and market situations and of the labour contract, as well as details of employment status (six levels, in increasing deprivation order: managerial and professional occupations, intermediate occupations, small employers and own account workers, lower supervisory and technical occupations, semi-routine occupations, other) [27]. Educational qualification was categorised based on the highest educational attainment and competence according to the National Vocational Qualification (NQV) criteria [28] (eight levels, in increasing deprivation order: NVQ4/NVQ5/Degree, higher education, NVQ3/GCE A Level, NVQ2/GCE O Level, NVQ1/CSE other grade, foreign/other qualification, no qualification and full-time student).

### Concentration index

The Erreygers and the Wagstaff concentration index are two popular measures of socioeconomic inequality in health [29]. While both indices can quantify the level of inequality in health outcomes across different groups, the choice between using the Erreygers corrected concentration index (CCI) and the Wagstaff concentration index depends on the normative priorities ought to be considered and the specific context in which the index is applied. The implications of the bounded nature of the outcome variable for the concentration index have been thoroughly discussed in the literature, with a few correction methods being proposed [30, 31]. In terms of difference in their mathematical properties, the CCI places greater weight on the health of the most deprived, whereas the Wagstaff concentration index gives an equal weight to the entire inequality distribution [32].

We employed the CCI to measure absolute inequalities in excess weight across the three dimensions of socio-economic deprivation specified above. We chose the CCI as it has been recommended for cardinal health variables [30] such as BMI, and it has been widely used in previous studies on excess weight [33–37], so it enables comparison of findings. Furthermore, the CCI is in principle normatively better aligned with and more suitable for informing public health policymakers who aim to prioritize improving the health of the most deprived groups. For completeness, we additionally performed a robustness check using the Wagstaff Concentration Index.

The CCI can be expressed formally [38] as follows:

$$\mathrm{CCI}=\frac{4 \times\upmu }{\mathrm{b}-\mathrm{a}}\times \frac{2 \times \mathrm{ cov }\left(\mathrm{yi},\mathrm{ ri }\right)}{\upmu }$$

where yi is the BMI measure for each individual (i), µ represents the mean BMI value, ri is the individual’s fractional rank along the socio-economic distribution of interest, cov denotes the covariance, and *a* and *b* are the lower and higher bounds of the excess weight measure. The CCI can range between -1 and 1, and a positive value indicates that the burden of excess weight is disproportionately borne by the most deprived individuals, and vice versa.

### Decomposition analysis

To quantify the independent effect of key factors contributing to the observed socio-economic deprivation-related inequalities in excess weight, we used the Shapley decomposition method [39]. Decomposition analyses were performed at the mean, as well as across the excess weight spectrum, that is considering inequalities in overweight (BMI >  = 25), obesity (BMI >  = 30) and morbid obesity status (BMI >  = 35). The Shapley method allowed us to evaluate how the explanatory variables independently contributed to the explained variance, and therefore assess their relative importance to the estimated inequalities. This method computes marginal effects by eliminating each covariate in sequence and then assigns to each factor the average of its marginal contribution in all its possible elimination sequences.

### Statistical analysis

We used summary statistics and graphical representations to describe socio-demographic characteristics and the distribution of BMI across the English and regional adult population. To test for differences in personal characteristics between sub-samples, we used independent sample t-tests or analysis of variance for continuous variables, as appropriate, and Pearson χ2 tests for categorical variables. An informal analysis of residuals was conducted for significant estimates of categorical variables with more than two levels.

A forward stepwise approach for model specification was employed, with three models being built progressively. Specification 1 included intrinsic individual characteristics, namely, region of residence, age, gender and ethnic background. This specification was then enhanced by considering individual’s personal circumstances, namely, whether they had long-lasting limiting illness, their marital status and urbanicity (e.g., they lived in an urban area or not, specification 2). The full specification was further augmented by the socio-economic deprivation dimensions. Model selection was based on the Bayesian information criterion [40]. All analyses were performed using STATA 16 software [41] and we used the command svyset to account for survey weights.

## Results

Table 1 shows that, in 2019, the English adult population had an average BMI of 27.9 (0.08). One third were at a healthy weight, while 37.5% were overweight and one in ten adults had a BMI of at least 35 (obesity I and II). Adults were uniformly distributed across IMD quintiles, around 40% held at least a higher education and had either managerial or intermediate occupations. Significant differences emerged across regions. The West Midlands had the highest average BMI (28.7 (0.27)), closely followed by the South-West and North-West regions, both with an average BMI of 28.5 (0.27). These differences in BMI were mostly driven by higher proportions of individuals living with obesity II and obesity III which made up 13.7% in the West Midlands, compared to almost half (7.3%) that in the London area. In terms of age, London had a comparatively younger population, with 44.5% aged between 20 and 39 years old, compared to 26.7% of 20–39-year-olds in the West Midlands.

**Table 1:** Distribution of excess weight and socio-demographic characteristics of the English adult population and regions—proportions (%) and standard deviations (in parenthesis) unless otherwise stated

	**England**	**North-East**	**North-West**	**Yorkshire and Humber**	**East Midlands**	**West Midlands**	**East of England**	**London**	**South East**	**South West**
**BMI score**										
Mean	28.03	28.62	28.36	28.18	28.27	28.61	27.90	27.16	27.41	28.40
	(5.7)	(6.0)	(6.0)	(5.9)	(5.7)	(5.8)	(5.4)	(5.3)	(5.6)	(5.6)
**BMI category**										
Underweight	0.01	0.01	0.00	0.00	0.01	0.01	0.01	0.02	0.01	0.01
	(0.1)	(0.1)	(0.1)	(0.1)	(0.1)	(0.1)	(0.1)	(0.1)	(0.1)	(0.1)
Healthy weight	0.31	0.28	0.29	0.34	0.30	0.26	0.31	0.35	0.35	0.28
	(0.5)	(0.4)	(0.5)	(0.5)	(0.5)	(0.4)	(0.5)	(0.5)	(0.5)	(0.4)
Overweight	0.38	0.34	0.39	0.36	0.36	0.39	0.39	0.37	0.38	0.39
	(0.5)	(0.5)	(0.5)	(0.5)	(0.5)	(0.5)	(0.5)	(0.5)	(0.5)	(0.5)
Obesity	0.30	0.37	0.31	0.30	0.33	0.34	0.29	0.26	0.26	0.32
	(0.5)	(0.5)	(0.5)	(0.5)	(0.5)	(0.5)	(0.5)	(0.4)	(0.4)	(0.5)
**Sex**										
Males	0.45	0.45	0.45	0.45	0.46	0.45	0.47	0.43	0.45	0.45
	(0.5)	(0.5)	(0.5)	(0.5)	(0.5)	(0.5)	(0.5)	(0.5)	(0.5)	(0.5)
Females	0.55	0.55	0.55	0.55	0.54	0.55	0.53	0.57	0.55	0.55
	(0.5)	(0.5)	(0.5)	(0.5)	(0.5)	(0.5)	(0.5)	(0.5)	(0.5)	(0.5)
**Age group**										
20–29 years	0.11	0.09	0.12	0.13	0.11	0.06	0.08	0.15	0.11	0.10
	(0.3)	(0.3)	(0.3)	(0.3)	(0.3)	(0.2)	(0.3)	(0.4)	(0.3)	(0.3)
30–39 years	0.17	0.14	0.18	0.21	0.17	0.16	0.20	0.21	0.14	0.14
	(0.4)	(0.3)	(0.4)	(0.4)	(0.4)	(0.4)	(0.4)	(0.4)	(0.4)	(0.3)
40–49 years	0.18	0.16	0.18	0.18	0.15	0.19	0.17	0.20	0.18	0.14
	(0.4)	(0.4)	(0.4)	(0.4)	(0.4)	(0.4)	(0.4)	(0.4)	(0.4)	(0.3)
50–59 years	0.18	0.21	0.19	0.15	0.18	0.19	0.17	0.19	0.17	0.20
	(0.4)	(0.4)	(0.4)	(0.4)	(0.4)	(0.4)	(0.4)	(0.4)	(0.4)	(0.4)
60–69 years	0.17	0.16	0.17	0.14	0.19	0.17	0.17	0.14	0.18	0.19
	(0.4)	(0.4)	(0.4)	(0.3)	(0.4)	(0.4)	(0.4)	(0.3)	(0.4)	(0.4)
70–79 years	0.14	0.16	0.11	0.13	0.15	0.15	0.16	0.10	0.15	0.16
	(0.3)	(0.4)	(0.3)	(0.3)	(0.4)	(0.4)	(0.4)	(0.3)	(0.4)	(0.4)
80 + years	0.06	0.08	0.05	0.05	0.05	0.07	0.06	0.03	0.06	0.07
	(0.2)	(0.3)	(0.2)	(0.2)	(0.2)	(0.3)	(0.2)	(0.2)	(0.2)	(0.3)
**Ethnicity**										
White	0.87	0.95	0.88	0.88	0.92	0.83	0.89	0.62	0.92	0.93
	(0.3)	(0.2)	(0.3)	(0.3)	(0.3)	(0.4)	(0.3)	(0.5)	(0.3)	(0.3)
Mixed	0.02	0.00	0.02	0.02	0.01	0.01	0.01	0.03	0.01	0.02
	(0.1)	(0.1)	(0.1)	(0.1)	(0.1)	(0.1)	(0.1)	(0.2)	(0.1)	(0.1)
Asian	0.08	0.04	0.08	0.06	0.05	0.13	0.06	0.21	0.06	0.03
	(0.3)	(0.2)	(0.3)	(0.2)	(0.2)	(0.3)	(0.2)	(0.4)	(0.2)	(0.2)
Black	0.03	0.00	0.02	0.02	0.01	0.02	0.03	0.10	0.01	0.01
	(0.2)	(0.0)	(0.1)	(0.2)	(0.1)	(0.1)	(0.2)	(0.3)	(0.1)	(0.1)
Other	0.01	0.00	0.00	0.02	0.01	0.00	0.00	0.04	0.00	0.00
	(0.1)	(0.1)	(0.1)	(0.1)	(0.1)	(0.0)	(0.1)	(0.2)	(0.1)	(0.1)
**Education**										
NVQ4/NVQ5/Degree or equiv	0.31	0.25	0.27	0.26	0.25	0.23	0.32	0.49	0.34	0.29
	(0.5)	(0.4)	(0.4)	(0.4)	(0.4)	(0.4)	(0.5)	(0.5)	(0.5)	(0.5)
Higher Ed. below degree	0.11	0.12	0.11	0.12	0.12	0.14	0.11	0.08	0.11	0.13
	(0.3)	(0.3)	(0.3)	(0.3)	(0.3)	(0.4)	(0.3)	(0.3)	(0.3)	(0.3)
NVQ3/GCE A Level or equiv	0.14	0.15	0.16	0.12	0.15	0.14	0.15	0.11	0.15	0.12
	(0.3)	(0.4)	(0.4)	(0.3)	(0.4)	(0.4)	(0.4)	(0.3)	(0.4)	(0.3)
NVQ2/GCE 0 Level or equiv	0.18	0.23	0.18	0.18	0.19	0.20	0.16	0.12	0.18	0.21
	(0.4)	(0.4)	(0.4)	(0.4)	(0.4)	(0.4)	(0.4)	(0.3)	(0.4)	(0.4)
NVQ1/CSE or equiv	0.04	0.04	0.04	0.05	0.02	0.04	0.03	0.02	0.04	0.04
	(0.2)	(0.2)	(0.2)	(0.2)	(0.2)	(0.2)	(0.2)	(0.1)	(0.2)	(0.2)
Foreign/Other	0.01	0.02	0.01	0.01	0.01	0.01	0.02	0.01	0.01	0.01
	(0.1)	(0.1)	(0.1)	(0.1)	(0.1)	(0.1)	(0.1)	(0.1)	(0.1)	(0.1)
No qualification	0.19	0.18	0.21	0.23	0.22	0.20	0.18	0.15	0.14	0.18
	(0.4)	(0.4)	(0.4)	(0.4)	(0.4)	(0.4)	(0.4)	(0.4)	(0.3)	(0.4)
Full-time student	0.02	0.01	0.03	0.02	0.02	0.02	0.02	0.03	0.03	0.03
	(0.2)	(0.1)	(0.2)	(0.2)	(0.1)	(0.1)	(0.1)	(0.2)	(0.2)	(0.2)
**Occupation**										
Managerial/professional	0.39	0.33	0.35	0.33	0.34	0.33	0.43	0.48	0.44	0.38
	(0.5)	(0.5)	(0.5)	(0.5)	(0.5)	(0.5)	(0.5)	(0.5)	(0.5)	(0.5)
Intermediate occupations	0.14	0.16	0.12	0.14	0.15	0.14	0.15	0.14	0.15	0.14
	(0.3)	(0.4)	(0.3)	(0.3)	(0.4)	(0.3)	(0.4)	(0.3)	(0.4)	(0.3)
Small employers/own account workers	0.11	0.09	0.09	0.09	0.10	0.09	0.12	0.10	0.11	0.16
	(0.3)	(0.3)	(0.3)	(0.3)	(0.3)	(0.3)	(0.3)	(0.3)	(0.3)	(0.4)
Lower supervisory/technical	0.07	0.07	0.05	0.09	0.08	0.09	0.07	0.04	0.06	0.06
	(0.3)	(0.3)	(0.2)	(0.3)	(0.3)	(0.3)	(0.3)	(0.2)	(0.2)	(0.2)
Semi-routine	0.28	0.34	0.35	0.32	0.31	0.34	0.23	0.21	0.21	0.26
	(0.4)	(0.5)	(0.5)	(0.5)	(0.5)	(0.5)	(0.4)	(0.4)	(0.4)	(0.4)
Other	0.02	0.01	0.03	0.02	0.02	0.02	0.01	0.03	0.02	0.01
	(0.1)	(0.1)	(0.2)	(0.2)	(0.1)	(0.1)	(0.1)	(0.2)	(0.1)	(0.1)
**Index of Multiple Deprivation**										
Most deprived: 34.42—85.46	0.20	0.27	0.35	0.33	0.21	0.28	0.10	0.17	0.07	0.06
	(0.4)	(0.4)	(0.5)	(0.5)	(0.4)	(0.4)	(0.3)	(0.4)	(0.3)	(0.2)
21.22—34.42	0.19	0.26	0.20	0.14	0.15	0.20	0.15	0.32	0.13	0.22
	(0.4)	(0.4)	(0.4)	(0.4)	(0.4)	(0.4)	(0.4)	(0.5)	(0.3)	(0.4)
13.74—21.22	0.20	0.14	0.15	0.20	0.21	0.23	0.24	0.21	0.19	0.21
	(0.4)	(0.3)	(0.4)	(0.4)	(0.4)	(0.4)	(0.4)	(0.4)	(0.4)	(0.4)
8.32—13.74	0.20	0.15	0.16	0.19	0.23	0.14	0.23	0.16	0.23	0.31
	(0.4)	(0.4)	(0.4)	(0.4)	(0.4)	(0.4)	(0.4)	(0.4)	(0.4)	(0.5)
Least deprived: 0.37—8.32	0.21	0.19	0.14	0.13	0.19	0.15	0.28	0.14	0.38	0.20
	(0.4)	(0.4)	(0.3)	(0.3)	(0.4)	(0.4)	(0.5)	(0.3)	(0.5)	(0.4)
**Observations**	6387	548	833	664	573	606	771	776	1020	596

*BMI* body mass index, *NVQ* National Vocational Qualification, *GCE* General Certificate of Education

With respect to socio-economic deprivation, in the London area, managerial and professional occupations and top educational qualifications were disproportionally represented compared to the rest of England, particularly relative to the North and the West Midlands regions. Even more heterogeneous distributions were found in terms of adults living in least deprived areas. The South and East of England showed a higher proportion of adults from the bottom IMD quintile, relative to the North and West regions, and in London, half of the population lived in neighbourhoods classed as highly deprived (4^th^ or 5^th^ IMD quintiles).

### Socio-economic inequalities in excess weight

A positive deprivation gradient in excess weight was found across the English adult population (i.e., the most deprived were more affected by excess weight) for all three socio-economic dimensions (Table 2), as measured by the CCI. Comparable results were found when the Wagstaff Concentration Index was used (Additional file 1). Educational qualification ranked first, as indicated by the highest CCI –0.090 (*p* < 0.01), closely followed by IMD at -0.050 (*p* < 0.01). At the regional level, different patterns emerged. In the West Midlands and London, occupational status played a more important role than IMD, and in the two Southern regions, occupational status did not pattern excess weight. By contrast, for the remaining regions (except the North West, where no socio-economic deprivation-related inequalities were found), only IMD played a significant role. When focusing only on obesity, inequality patterns were consistent in direction and broadly comparable in magnitude to those observed for excess weight, both for England and the individual regions.

**Table 2:** Erreygers Corrected concentration indices (CCI) of excess weight and obesity inequalities across the English adult population and regions

	**Region**	**IMD quintile**	**Occupational status**	**Educational qualification**
**Excess weight**	North East	-0.0498***	-0.0412***	-0.0796***
	North West	0.0516*	0.0356**	-0.0328***
	Yorkshire and The Humber	-0.0292***	-0.0026***	-0.0451***
	East Midlands	-0.0846***	-0.0192***	-0.0673***
	West Midlands	-0.0251***	-0.0602***	-0.0916***
	East of England	-0.0855***	0.0407**	-0.0256***
	London	-0.0471***	-0.0793***	-0.1516***
	South East	-0.1018***	-0.0773***	-0.0952***
	South West	-0.0349***	-0.0051***	-0.0651***
	England	-0.0498***	-0.0381***	-0.0896***
**Obesity**	North East	-0.0971***	-0.0894***	-0.0146***
	North West	-0.0157***	-0.0129***	-0.0556***
	Yorkshire and The Humber	-0.0906***	0.0016***	-0.0549***
	East Midlands	-0.1084***	-0.0772***	-0.0474***
	West Midlands	-0.1118***	-0.1003***	-0.1163***
	East of England	-0.0918***	-0.0080***	-0.0858***
	London	-0.0581***	-0.0952***	-0.1445***
	South East	-0.0762***	-0.0377***	-0.0991***
	South West	-0.0662***	-0.0082***	-0.0673***
	England	-0.0838***	-0.0576***	-0.0982***

^***^p < .01, ** *p* < .05, * *p* < 0.1; *IMD*  Index of Multiple Deprivation

Breaking down the results by gender, Table 3 shows that in most instances, the socio-economic inequalities in excess weight in England and at the regional level were either considerably higher or exclusively patterned among women. For example, in the North East [CCI: -0.119, *p* < 0.01], East Midlands [CCI: -0.156, *p* < 0.01], the South-West [CCI: -0.087, *p* < 0.01] and East of England [CCI: -0.136, *p* < 0.01], it was predominantly the IMD in women that played a significant role in patterning excess weight. And in the West Midlands, the index for occupational status [CCI: -0.117, *p* < 0.01] was significantly larger for women, then it was for men [CCI: 0.006, *p* < 0.01]. In Yorkshire and Humber, IMD [CCI: -0.143, *p* < 0.01] and occupational status [CCI: -0.07, *p* < 0.01] played a role only within women, whereas in London and the South East it was educational qualification that patterned excess weight in women. Conversely, in the North West, and similar to the all-gender regional analysis, none of the three socio-economic measures had an effect on inequality, in either gender.

**Table 3:** Erreygers corrected concentration indices (CCI) of excess weight inequalities across the English adult population and regions, by gender

	**Region**	**IMD quintile**	**Occupational status**	**Educational qualification**
**Men**	North East	0.0280**	0.0867*	-0.0861***
	North West	0.1001	0.0710*	0.0017***
	Yorkshire and The Humber	0.0927*	0.0734*	-0.0220***
	East Midlands	-0.0101***	0.0257**	-0.0727***
	West Midlands	-0.0045***	0.0064***	-0.0876***
	East of England	-0.0366***	0.0671*	0.0308**
	London	0.0453**	-0.0758***	-0.0949***
	South East	-0.0798***	-0.0262***	-0.0278***
	South West	0.0293**	0.0359**	-0.0501***
	England	0.0020	0.0005	-0.0593***
**Women**	North East	-0.1190***	-0.1561***	-0.0850***
	North West	0.0126**	0.0327**	-0.0618***
	Yorkshire and The Humber	-0.1433***	-0.0665***	-0.0645***
	East Midlands	-0.1565***	-0.0777***	-0.0716***
	West Midlands	-0.0464***	-0.1168***	-0.1046***
	East of England	-0.1364***	0.0008***	-0.0963***
	London	-0.1267***	-0.0879***	-0.2014***
	South East	-0.1228***	-0.1338***	-0.1927***
	South West	-0.0870***	-0.0268***	-0.0719***
	England	-0.0987***	-0.0749***	-0.1248***

^***^p < .01, ** *p* < .05, * *p* < 0.1; IMD = Index of Multiple Deprivation

### Factors contributing to inequalities in excess weight

Neighbourhood-level deprivation (IMD) accounted for 33.9% of the total variance in inequality, after adjusting for region of residence, age, gender and ethnicity (specification 1, Tables 4 and 5). By further controlling for respondent characteristics (long-lasting illness status, marital status and urbanicity), the contribution of IMD to the inequality in excess weight decreased by almost a half, to 19%. Comparable adjustment patterns were observed for occupational status and educational qualification-related inequalities.

**Table 4:** (part 1) Shapley decomposition of factors contributing to excess weight inequalities (continued in the next page)

	**Neighbourhood-level deprivation**						**Occupational status**					
	**Specification 1**			**Specification 2**			**Specification 1**			**Specification 2**		
	DI	0.219		DI	0.300		DI	0.197		DI	0.283	
	Shapley Value	contrib. (%)	*p*-value	Shapley Value	contrib. (%)	*p*-value	Shapley Value	contrib. (%)	*p*-value	Shapley Value	contrib. (%)	*p*-value
**IMD**	0.014	40.67	0.000	0.013	23.52	0.000	-	-	-	-	-	-
**Region**	0.002	4.27	0.173	0.001	2.49	0.225	0.002	7.59	0.011	0.002	3.93	0.023
**Age**	0.002	5.48	0.010	0.004	7.52	0.000	0.002	9.09	0.001	0.005	10.38	0.000
**Gender**	0.015	42.47	0.000	0.013	24.27	0.000	0.016	59.34	0.000	0.014	30.40	0.000
**Ethnic background**	0.003	7.12	0.000	0.002	3.73	0.000	0.002	5.91	0.004	0.001	2.86	0.011
**Long-lasting illness**	-	-	-	0.021	37.96	0.000	-	-	-	0.020	43.06	0.000
**Marital status**	-	-	-	0.000	0.20	0.324	-	-	-	0.000	0.24	0.345
**Urbanicity**	-	-	-	0.000	0.31	0.921	-	-	-	0.000	0.59	0.387
**Occupational status**	-	-	-	-	-	-	0.005	18.07	0.000	0.004	8.53	0.000
**Educational qualification**	-	-	-	-	-	-	-	-	-	-	-	-
**Observations**	4,335			4,335			4,219			4,216		

*IMD* Index of Multiple Deprivation, *DI* Dissimilarity Index. Specification approach described in subsection “ Statistical analysis”

**Table 5:** (part 2) Shapley decomposition of factors contributing to excess weight inequalities

	**Educational qualification**						**Specification 3**		
	**Specification 1**			**Specification 2**					
	DI	0.203		DI	0.289		DI	0.307	
	Shapley Value	contrib. (%)	*p*-value	Shapley Value	contrib. (%)	*p*-value	Shapley Value	contrib. (%)	*p*-value
**IMD**	-	-	-	-	-	-	0.011	19.13	0.000
**Region**	0.002	7.61	0.007	0.002	4.01	0.015	0.001	2.14	0.276
**Age**	0.004	13.33	0.000	0.006	12.92	0.000	0.005	8.33	0.000
**Gender**	0.015	52.59	0.000	0.013	27.38	0.000	0.014	24.66	0.000
**Ethnic background**	0.002	5.82	0.002	0.001	2.84	0.007	0.002	3.23	0.002
**Long-lasting illness**	-	-	-	0.021	42.74	0.000	0.019	33.85	0.000
**Marital status**	-	-	-	0.000	0.17	0.411	0.000	0.32	0.222
**Urbanicity**	-	-	-	0.000	0.53	0.458	0.000	0.29	0.918
**Occupational status**	-	-	-	-	-	-	0.002	3.97	0.051
**Educational qualification**	0.006	20.65	0.000	0.005	9.40	0.000	0.002	4.09	0.160
**Observations**	4,324			4,321			4,203		

*IMD* Index of Multiple Deprivation, *DI* dissimilarity index. Specification approach described in subsection “Statistical analysis”

The fully adjusted model (specification 3) showed that IMD independently accounted for 15% of the total variance, more than occupational status and educational qualification combined (6.5% and 7.5% respectively). Of interest, these results also indicated that over half (55.5%) of the variation in excess weight across England was explained by a person’s long-lasting illness status and gender together. Further heterogeneity analyses showed that these relative contributions to inequalities differed between genders. In particular, a long-lasting illness status explained almost half of the variance (48.6%) in women, while only 27% in men. By contrast, IMD showed a relative contribution of 25.6% in men and 15.6% in women.

### Factors contributing to inequalities across the excess weight spectrum

On closer inspection (Table 6), the relative contributions measured ‘at the mean’ (Tables 4 and 5) were found to be inconsistent across the excess weight spectrum. Neighbourhood-level deprivation, a long-lasting illness status and educational qualification played a greater role in contributing to inequalities in obesity than in overweight status. By contrast, age and gender, that when combined contributed to over 60% in overweight status, explained less than 20% of the variance in obesity status.

**Table 6:** Shapley decomposition of factors contributing to inequalities across the excess weight spectrum

	**Overweight**			**Obese**			**Class II and III obese**		
	DI	0.056		DI	0.070		DI	0.073	
	Value	contrib. (%)	*p*-value	Value	contrib. (%)	*p*-value	Value	contrib. (%)	*p*-value
**IMD**	0.011	19.13	0.000	0.009	12.38	0.002	0.002	3.38	0.378
**Region**	0.001	2.14	0.276	0.001	0.88	0.885	< 0.001	0.55	0.886
**Age**	0.005	8.33	0.000	0.013	18.64	0.000	0.041	55.47	0.000
**Gender**	0.014	24.66	0.000	0.015	21.02	0.000	0.013	17.25	0.010
**Ethnic background**	0.002	3.23	0.002	0.001	1.48	0.092	< 0.001	0.24	0.664
**Long-lasting illness**	0.019	33.85	0.000	0.025	36.59	0.000	0.014	19.66	0.000
**Marital status**	< 0.001	0.32	0.222	< 0.001	0.68	0.256	0.001	0.71	0.755
**Urbanicity**	< 0.001	0.29	0.918	< 0.001	0.48	0.947	< 0.001	0.16	0.714
**Occupational status**	0.002	4.09	0.160	0.002	3.56	0.315	< 0.001	0.53	0.938
**Educational qualification**	0.002	3.97	0.051	0.003	4.31	0.062	0.001	2.05	0.192
**Observations**	4,203			1,880			671		

*IMD* Index of Multiple Deprivation, *DI* dissimilarity index

Gender-related heterogeneity analyses revealed that contribution patterns of key factors were consistent in direction between genders, but their relative contributions varied considerably in magnitude, particularly in terms of overweight status. Age alone accounted for 59.3% of the variance in overweight status in men, whereas in women this factor only contributed to 31.5%. On the other hand, a long-lasting illness status and IMD explained 10% and 1% of the inequality in men, whereas in women they played a much greater role at 31.2% and 14.6%, respectively.

## Discussion

### Main findings

This study provides up-to-date estimates of the socio-economic inequalities in excess weight across the English adult population and identifies key factors contributing to these inequalities, both on average and across the excess weight spectrum. Systematic differences were found with the probability of having excess weight or living with obesity across socio-economic groups in England. Adults with a lower educational qualification, lower occupational status or living in more deprived neighbourhoods were consistently more affected by excess weight, relative to their less deprived counterparts. However, there was a large heterogeneity between regions, with the three measures of deprivation playing different, and in some instances no role, in patterning excess weight across socio-economic groups. Marked differences were also found between genders, with socio-economic inequalities in excess weight being either considerably higher or exclusively patterned among women.

A long-lasting illness status was found to be the single strongest independent factor contributing to excess weight inequalities in the English adult population. This was especially the case among women and towards the right tail of the excess weight spectrum. Although the patterns of key factors that contributed to excess weight along the spectrum were consistent in direction in both men and women, they differed markedly in the degree to which they contributed. Most of these differences were at the overweight end of the spectrum, where neighbourhood-level deprivation played a dominant role particularly among men.

Our findings underscore the importance of considering factors such as gender and multimorbidity when developing policies to address overweight and obesity. From a public health perspective, this calls for considering the unique needs and experiences of different populations and designing targeted interventions to address the specific barriers to healthy behaviours faced by these populations, including limited access to healthy food options or reduced physical activity opportunities (due, for example, to time restrictions caused by care responsibilities). Moreover, examining the relationship between area-level and individual-level measures and overweight enabled us to provide some insights into the complex interplay between individual and environmental factors in shaping excess weight inequalities. This has the potential to inform targeted policies and interventions that address relevant individual and environmental factors to promote healthy weight and reduce health inequalities at the local level. At the area-level, deprived neighbourhoods often lack adequate access to healthy food options and opportunities for physical activity, which can contribute to higher rates of obesity. At the individual-level, low-income individuals may face financial barriers to accessing healthy food and opportunities for physical activity and may also experience greater stress and mental health challenges which can contribute to weight gain.

Similarly, examining gender differences in excess weight inequalities is relevant from a public health perspective, as the risk of developing a chronic disease, from the same level of obesity, appears to be significantly different between gender groups. An example is cancer, whereby women are at a greater risk than men, as evidenced by the study by Steele et al. (2017), who found that – including gender-specific conditions such as endometrial and ovarian cancer for women and prostate cancer for men—55% of all cancer diagnosis in women in 2014 in the US were linked to excess weight, whereas for men this proportion was less than a half, 24% [42]. Public health interventions may be effective if an effective targeting of the underlying causes of their excess weight is carried out. Furthermore, from a societal point of view, targeted interventions for women and individuals with long-lasting illnesses can have both short-term and long-term economic benefits, by reducing healthcare costs and increasing workforce productivity.

Our findings confirm that marked regional differences exist, particularly between the North and the South of England. These do not only emerge in terms of gradient in average obesity levels, but in the socio-economic factors most markedly driving obesity inequalities at the local level. While a number of individual-level controls have been accounted for in our analysis, the presented estimates may be also explained by macro level factors such differences in regional economic activity and productivity which characterise England [43]. This further highlights the need for obesity inequality-tacking policy to be carefully tailored to the specific needs and characteristics of the local population and socio-economic environment.

Finally, behaviours such as physical activity [44], unbalanced diets [45], and alcohol consumption [46] play a role in shaping social inequalities in obesity. Lower income and education levels are associated with higher physical inactivity levels and consumption of energy-dense and low-nutrient foods. Furthermore, evidence suggests excessive alcohol consumption is more common among lower socio-economic status individuals [47]. These health behaviours are also closely related to area-level characteristics including access to healthy food options and green space.

### Comparison with previous studies

Our study adds to the growing evidence highlighting a social gradient in excess weight in England, and more generally, in developed countries where lower socioeconomic status has been systematically associated with higher excess weight [48–51]. For instance, Mireku & Rodriguez (2019) analysed a nationally representative cohort of UK adolescents (the Millennium Cohort Study) and found that risk of obesity, overweight, and adiposity increased with decreasing family income quintiles [33]. Zhang and Wang (2004) used the concentration curve and concentration index to study the socioeconomic disparity in obesity among American adults aged 18–60 years and found a reverse association between socio-economic status and obesity among both white women and men, and particularly in young white women [35]. They argue that although minority groups do not necessarily have a higher socioeconomic status inequality in obesity than white individuals, they are more vulnerable to obesity. Molarius et al. (2000) measured the association between education level and BMI in adults between 36 and 64 years old in 26 countries using the WHO MONICA Project database [36]. They found that low education was associated with a higher BMI in about half of the males and in almost all of the females studied, and the existence of an increasing tendency in this association across the 10 years of the study.

The stronger positive association between socio-economic deprivation and obesity among women compared with men has also been observed in previous studies [35, 37, 52–54]. Madden (2013) found a steeper socioeconomic gradient of obesity among women than men in Ireland. However, the authors noted the gap between the gradients had narrowed between 2002 and 2007, because of increasing obesity amongst lower income men and higher income women [53]. Zhang and Wang (2004) also found gender differences in the direction of the association between SES and obesity; while socio-economic status and obesity always held an inverse association among females, the association was positive in some male minority groups [35]. Pudrovska et al. (2014) found that socioeconomic disadvantage at age 18 is related to higher BMI and risk of obesity at age 54, and that this relationship is stronger for women than it is for men [54]. Most importantly, the authors suggest that that body mass and socio-economic position are simultaneously antecedents and consequences of each other over the life course, creating what they call a “reciprocal chain of disadvantage of heavier body mass and lower socio-economic status” that is stronger among women compared to men.

### Strengths and limitations

To the best of our knowledge, this is the first study that provides a comparative national and regional-level assessment of the inequalities related to multiple dimensions of socio-economic deprivation in the English adult population. We used two individual-level (occupational status and educational qualification) and a neighbourhood-level (IMD) socio-economic measure, which are widely and routinely used to inform national and local authority decision making in England. The concentration index has been increasingly used to quantify the degree of socio-economic-related inequality in health variables, including excess weight. However, it is worth noting that this is a summary measure of inequality which can take the value of zero if there is not a clear gradient between categories of the ranking variable (no concentration), even when there is inequality between the bottom and the top categories [55].

We acknowledge that other dimensions of socio-economic deprivation could also have been selected, notably income. However, our choice was motivated by data availability. The only information available within HSE regarding income was the level (tertile or quintile) of household income. Our focus was particularly on comparing deprivation-related inequalities and, due to nonnegligible differences in purchasing power between English regions [56], using the existing income level variables would have inevitably induced bias in the comparative analyses. In addition, when considering the inclusion of this dimension only for the national level analysis, we decided to exclude it as the information on income level was missing for a sizeable proportion of respondents (32%), which would have otherwise precluded our ability to draw any meaningful conclusions with a reasonable level of confidence. Furthermore, recent evidence has emerged showing that income may account only for a minor proportion of the socio-economic gradient in excess-weight [57].

We applied a series of survey weights and correction methods to address the issues of sampling and selection bias due to missing data – which are common in health studies yet are often overlooked [58] – to ensure the sample and therefore the results were representative of the adult population. While these are principled methods, their validity crucially depends on the plausibility of the underlying assumption regarding the causes of missingness – more specifically, missing at random – which is untestable. However, in the absence of information regarding the selection process, these present a preferable choice over ad hoc methods, such as complete case analysis [59].

Our findings are based on a cross-sectional analysis of survey data collected over one year. It does not attempt to make any causal claims about the link between the explanatory variables and excess weight measures or to disentangle cohort effects. Furthermore, a lack of data, particularly on covariates such as those related to a neighbourhood-obesogenic environment [14], limited the extent of statistical analysis for adequately addressing potential confounding, as well as the explanatory power of the models. We employed a model selection approach comparable to previous similar studies [16, 18] and a robust decomposition method (Shapley method). Unlike other decomposition methods, with this method we were able to estimate the independent contribution that each of the explanatory factors made to the variance in inequality. However, this method particularly requires large samples [38] and therefore limited our ability to pursue a disaggregated analysis (at a regional level) of the factors contributing to inequality in excess weight accordingly. Nonetheless, we explored how key factors contributed to the studied inequalities across the excess weight spectrum and between genders, hence potentially providing policy makers with evidence to inform the design of future inequity-curbing interventions in England.

## Conclusions

A social gradient, patterned by multiple dimensions of socio-economic deprivation, exists in the English adult population. The most socio-economically disadvantaged groups are affected the most by excess weight and obesity. Large heterogeneity exists between regions and genders, both in terms of prevalence of excess weight inequalities and their key contributing factors. In answering the posed research question, we recommend that policy makers pay careful consideration as to which socio-economic dimensions of deprivation and contributing factors do matter the most in their decision-making context and use that information to help prioritise and design inequity-curbing interventions. This will ensure that a further widening of the existing unfair and unjust inequalities in excess weight, and therefore loss of population health and well-being is avoided.

## Supplementary Information

**Additional file 1. **Wagstaff concentration index analyses. **Table A** Wagstaff concentration indices of excess weight and obesity inequalities across the English adult population and regions. **Table B** Wagstaff concentration indices of excess weight inequalities across the English adult population and regions, by gender

## Data Availability

The datasets analysed during the current study are available at https://digital.nhs.uk/data-and-information/publications/statistical/health-survey-for-england/2019.
