# Supplementary material for: Socio-economic accounting of inequalities in excess weight: a population-based analysis
Source: BMC Public Health. 2023 Apr 20;23:721. doi: 10.1186/s12889-023-15592-0 (PMC10116779; doi:10.1186/s12889-023-15592-0)
Supplement: Supplementary file 1 — Additional file 1. Wagstaff concentration index analyses. Table A Wagstaff concentration indices of excess weight and obesity inequalities across the English adult population and regions. Table B Wagstaff concentration indices of excess weight inequalities across the English adult population and regions, by gender [file 12889_2023_15592_MOESM1_ESM.docx]

# **Additional file 1 – Wagstaff concentration index analyses**

**Table A** Wagstaff concentration indices of excess weight and obesity inequalities across the English adult population and region

|  | **Region** | **IMD quintile** | **Occupational status** | **Educational qualification** |
| --- | --- | --- | --- | --- |
| **Excess weight** | North East | -0.0606*** | -0.0510*** | -0.0970*** |
|  | North West | 0.0626* | 0.0435** | -0.0397*** |
|  | Yorkshire and The Humber | -0.0319*** | -0.0028*** | -0.0494*** |
|  | East Midlands | -0.0987*** | -0.0228*** | -0.0784*** |
|  | West Midlands | -0.0326*** | -0.0782*** | -0.1186*** |
|  | East of England | -0.0975*** | 0.0465** | -0.0292*** |
|  | London | -0.0494*** | -0.0833*** | -0.1591*** |
|  | South East | -0.1096*** | -0.0841*** | -0.1025*** |
|  | South West | -0.0436*** | -0.0065*** | -0.0813*** |
|  | England | -0.0567*** | -0.0437** | -0.1020*** |
| **Obesity** | North East | -0.1052*** | -0.0960*** | -0.0159*** |
|  | North West | -0.0182*** | -0.0150*** | -0.0646*** |
|  | Yorkshire and The Humber | -0.1088*** | 0.0019*** | -0.0659*** |
|  | East Midlands | -0.1231*** | -0.0871*** | -0.0539*** |
|  | West Midlands | -0.1221*** | -0.1103*** | -0.1272*** |
|  | East of England | -0.1145*** | -0.0099*** | -0.1070*** |
|  | London | -0.0809*** | -0.1322*** | -0.2007*** |
|  | South East | -0.0998*** | -0.0491*** | -0.1299*** |
|  | South West | -0.0758*** | -0.0094*** | -0.0770*** |
|  | England | -0.1008*** | -0.0691*** | -0.1182*** |

**Notes:** *** p<.01, ** p<.05, * p<0.1; IMD= Index of Multiple Deprivati

**Table B** Wagstaff concentration indices of excess weight inequalities across the English adult population and regions, by gender

|  | **Region** | **IMD quintile** | **Occupational status** | **Educational qualification** |
| --- | --- | --- | --- | --- |
| **Men** | North East | 0.0377** | 0.1154 | -0.1159*** |
|  | North West | 0.1493 | 0.1078 | 0.0025*** |
|  | Yorkshire and The Humber | 0.1061 | 0.0842* | -0.0252*** |
|  | East Midlands | -0.0130*** | 0.0332** | -0.0934*** |
|  | West Midlands | -0.0068*** | 0.0096*** | -0.1318*** |
|  | East of England | -0.0431*** | 0.0807* | 0.0362** |
|  | London | 0.0493** | -0.0831*** | -0.1035*** |
|  | South East | -0.0929*** | -0.0316*** | -0.0324*** |
|  | South West | 0.0410** | 0.0499** | -0.0701*** |
|  | England | 0.0025 | 0.0006 | -0.0734*** |
| **Women** | North East | -0.1347*** | -0.1827*** | -0.0962*** |
|  | North West | 0.0135** | 0.0351** | -0.0667*** |
|  | Yorkshire and The Humber | -0.1517*** | -0.0706*** | -0.0685*** |
|  | East Midlands | -0.1710*** | -0.0871*** | -0.0781*** |
|  | West Midlands | -0.0542*** | -0.1372*** | -0.1220*** |
|  | East of England | -0.1513*** | 0.0009*** | -0.1068*** |
|  | London | -0.1298*** | -0.0898*** | -0.2061*** |
|  | South East | -0.1259*** | -0.1373*** | -0.1977*** |
|  | South West | -0.1000*** | -0.0311*** | -0.0825*** |
|  | England | -0.1059*** | -0.0806*** | -0.1338*** |

**Notes:** *** p<.01, ** p<.05, * p<0.1; IMD= Index of Multiple Deprivation
